# Assessing Muscle Mass in the Orthopedic Clinical Setting: Application of the Ultrasound Sarcopenia Index in Elderly Subjects with a Recent Femoral Fracture

**DOI:** 10.3390/nu16050711

**Published:** 2024-02-29

**Authors:** Luca Di Lenarda, Alex Buoite Stella, Chiara Ratti, Luca Ruggiero, Monica Bernard, Luisa Priscamaria Cavarzerani, Gianluca Canton, Luigi Murena

**Affiliations:** 1 Orthopaedics and Traumatology Unit, Department of Medicine, Surgery and Health Sciences, Azienda Sanitaria Universitaria Giuliano Isontina (ASUGI), Cattinara University Hospital, University of Trieste, Strada di Fiume 447, 34149 Trieste, Italy; lucadilenarda@gmail.com (L.D.L.); chiara.ratti@asugi.sanita.fvg.it (C.R.); luca.ruggiero@studenti.units.it (L.R.); monica.bernard@studenti.units.it (M.B.); gcanton@units.it (G.C.); lmurena@units.it (L.M.); 2 Department of Medicine, Surgery and Health Sciences, University of Trieste, Strada di Fiume 447, 34149 Trieste, Italy; 3 Single-Cycle Master’s Degree Course in Medicine and Surgery, Department of Medicine, Surgery and Health Sciences, University of Trieste, Strada di Fiume 447, 34149 Trieste, Italy; luisapriscamaria.cavarzerani@studenti.units.it

**Keywords:** sarcopenia, femur fracture, frailty, muscle, feasibility, traumatology

## Abstract

Background: Sarcopenia poses a risk factor for falls, disability, mortality, and unfavorable postoperative outcomes. Recently, the Ultrasound Sarcopenia Index (USI) has been validated to assess muscle mass, and this study aimed to apply the USI in the clinical setting. Methods: This prospective observational study included 108 patients aged >65 years, hospitalized for proximal femoral traumatic fracture. Patients were divided into two groups based on anamnestic data: patients with independent walking (IW) and patients requiring walking aid (WA) before admission. All the participants received an ultrasound examination. Other parameters evaluated were handgrip strength, limb circumferences, nutrition (MNA), and activity of daily living (ADL) scores. Results: Fifty-six IW patients (83 ± 6 y; 38 females) and 52 WA patients (87 ± 7 y; 44 females) were recruited. The USI was significantly higher in the IW group compared to the WA group (*p* = 0.013, Cohen’s *d* = 0.489). Significant correlations were found between the USI and other sarcopenia-associated parameters, such as handgrip strength, MNA, ADLs, other muscle ultrasound parameters, and limb circumferences. Conclusion: The application of the USI in the orthopedic surgery setting is feasible and might support the diagnosis of sarcopenia when combined with other measures of strength and function.

## 1. Introduction

Sarcopenia is defined as the loss of muscle mass in the elderly, which is an independent risk factor for falls, disability, postoperative complications, and mortality [1]. Typically, sarcopenia is the result of a complex bone–muscle interaction in the context of chronic disease and aging. To obtain a correct diagnosis, two of the three following criteria are required: low skeletal muscle mass, inadequate muscle strength, inadequate physical performance [2]. From an epidemiologic standpoint, about 44% of elderly who undergo orthopedic surgery and up to 24% of all patients between 65 and 70 years old are sarcopenic [3]. Within orthopedic surgery, sarcopenia has been observed as a risk factor for unfavorable postoperative outcomes, especially in the emergency setting. However, due to heterogeneity in studies and multiple methods utilized to evaluate sarcopenia, it is difficult to outline guidelines [3].

Even though there are plenty of tools for evaluating muscle mass in the clinical setting, these are often expensive and not practical, and for these reasons, they are scarcely used preoperatively [4]. Muscle ultrasound (US) has been suggested to be feasible in clinical practice, providing quantitative measures of muscle architecture in sports medicine, geriatrics, and other medical disciplines [5,6,7,8,9]. In 2003, Narici et al. reported that the loss of muscle mass associated with sarcopenia entails not only a decrease in muscle cross-sectional area and volume but also alterations in the spatial arrangement of muscle fibers within the muscle [specifically, fiber fascicle length (Lf), pennation angle (θ), and muscle thickness (Tm)], known as “muscle architecture” [4]. With the aid of US, it was possible to identify, for several locomotor muscles [10,11], that the key parameters of muscle architecture were significantly altered in sarcopenic muscle. In particular, as muscle volume, cross-sectional area, and Tm decrease with aging, fiber Lf and θ also become smaller. This spatial rearrangement of muscle fibers is expected to reflect a change in sarcomere number [12]: a decrease in fascicle length predicts a loss of sarcomeres in series [13,14], and a decrease in θ predicts a loss of sarcomeres arranged in parallel [15].

Recently, based on this knowledge, the Ultrasound Sarcopenia Index (USI) was validated based on using US to measure the ratio between the thickness of the vastus lateralis (VL) and the length of its fascicles [16]. An advantage of using a marker based on anatomical ratio rather than on absolute values is its independence from sex and body dimensions, as Lf and θ (and thus Tm) have been shown to be greater in men than women because of the difference in body height and body mass [17]. This procedure has many advantages: rapid execution compared to magnetic resonance imaging (MRI), being portable, low cost, presenting good reliability and reproducibility when performed by trained personnel [18,19,20], and no radiation compared to dual-energy X-ray absorptiometry (DXA). Therefore, it might be hypothesized that elderly people being admitted to an orthopedics/traumatology unit with a recent femur fracture might be characterized by sarcopenia and that the USI might help to detect it. In addition, it might identify those with anamnestic independent or assisted walking capacity.

Therefore, the primary aim of this study was to apply the USI to elderly patients hospitalized with a recent proximal femur fracture requiring orthopedic surgery, comparing those with independent walking (IW) with those who reported using walking aids (WAs) before the fracture. A secondary aim was to evaluate if correlations were present between the USI and other measures that are typically collected in the clinical practice in this population, such as handgrip strength, nutrition status, and activity of daily living (ADL) scores, as well as other anthropometrical measures such as upper and lower limb circumferences and skeletal muscle US measures.

## 2. Materials and Methods

### 2.1. Study Design and Participants

For this prospective cohort observational study, all the patients admitted from 26 October 2022 to 2 February 2023 to the orthopedics and traumatology unit of a university hospital with a diagnosis of proximal femur fracture were evaluated for inclusion and exclusion criteria. To be included, individuals from both sexes, ≥65 years old, with a traumatic proximal femur fracture (PFF) who were hospitalized and underwent orthopedic surgery for treatment of said fracture were considered. We excluded from our data sample patients who were previously bedridden or in a wheelchair; patients who had eating disorders; polytrauma patients or patients with multiple fractures; patients who had neurological disorders with peripheral neuropathy, muscle disorders, neurodegenerative diseases, and non-age-related bone disorders; patients who had endocrinological disorders with possible muscle or bone involvement; patients who had a history of tumoral disorders with possible bone involvement; and patients currently undergoing tumoral treatment. We also excluded patients who underwent orthopedic surgery in the previous two years that significantly impaired their mobility (e.g., total knee replacement in either leg, long bone fracture of the leg, contralateral proximal femur fracture) and patients with poorly controlled congestive heart failure or any other condition that could affect limb thickness. Based on the anamnestic evaluation, included participants were then categorized as independent walking (IW) or walking aid (WA) according to the reported needs to use any walking aid before hospital admission. All participants or their legal guardians signed an informed consent form, and the study was approved by the local ethical committee (122/2022). All procedures were performed according to the principles of the Declaration of Helsinki, and the Strengthening the Reporting of Observational Studies in Epidemiology (STROBE) guidelines were followed [21].

### 2.2. Data Collection and Calculated Measures

All data were collected within 48 h from admission in our unit, including patients’ demographics, anamnestic mini nutritional assessment (MNA) and ADL scores and clinical characteristics, anthropometrics (limb circumferences), handgrip strength, and muscle US. The MNA and ADL scores were calculated after direct communication with the patient and with the help of a relative if necessary. The MNA test is composed of some brief questions and simple measurements including (i) body mass, height, and body mass loss; (ii) lifestyle, medication, and mobility questions; (iii) dietary questions such as number of meals, food and fluid intake, and autonomy of feeding; and (iv) self-perception of health and nutrition. The collected data provide a score that in the elderly classifies those with adequate nutrition (MNA ≥ 24), at risk of malnutrition (MNA between 17.0 and 23.5), with protein–calorie malnutrition (MNA < 17) [22]. The index of the Independence in Activities of Daily Living scale was used to assess pre-fracture ADLs: this instrument investigated different aspects of daily living, and a score between 0 and 2 is given regarding the ability to perform these 6 activities, with a maximum score of 12 indicating optimal ADL independence [23]. All the assessments were performed by the same investigator on the limb without the fracture and on the dominant upper limb. 

Limb circumferences were measured with a tape at the points of maximum circumference between shoulder and elbow (arm), elbow and wrist (forearm), hip and knee (thigh), and knee and ankle (leg). The handgrip strength of the dominant limb was assessed with a portable dynamometer (K-force grip, Kinvent, Italy) according to a standardized protocol [8]. The shoulder on the dominant side was adducted, the elbow positioned in 90° flexion, and the wrist in a neutral position. As described by Lupton-Smith et al., the patient should be upright, with their knees and hips at 90° and with back support [8]. In patients with a proximal femur fracture, this position was not possible; therefore, handgrip measurements were performed with the bed reclined at 45°, which we believe was the best compromise to avoid making the patient feel pain and to collect the most realistic result possible. There were three separate measures of a duration of 5 s each, allowing 1 min of rest between each trial. 

**Figure 1:**
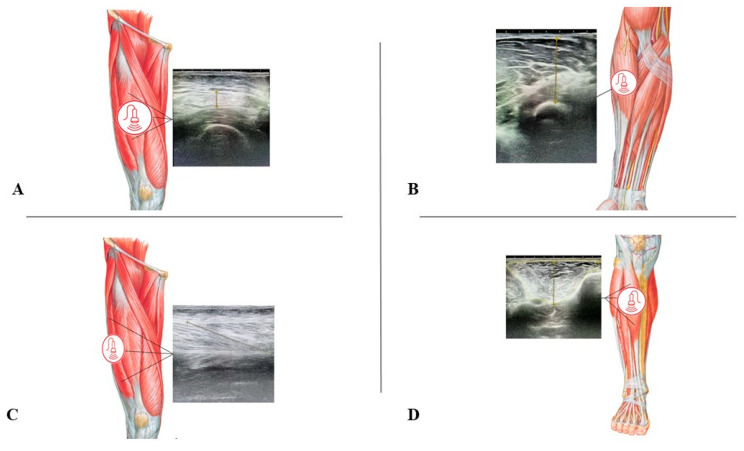
Representation of the ultrasound assessment procedure on the non-injured lower limb (**A**) rectus femoris, (**C**) vastus lateralis, and (**D**) tibialis anterior and the dominant limb (**B**) forearm muscle.

US measurements were then performed to assess several muscle architecture parameters, as previously suggested [16]. A digital ultrasound device (Samsung HS60A, Republic of Korea) was used, with a 3–14 MHz linear probe optimized for muscular evaluation, as previously described [16]. All images were collected twice by two trained investigators with previous experience in US research. Before the study, the investigators who performed US assessments were required to obtain high-quality ultrasound images from the vastus lateralis (VL) in a similar population, with higher inter-day reliability tested in repeated examinations performed two days apart. A third experienced US investigator judged the quality of the images, and a high inter-day reliability was defined as an intraclass correlation coefficient (ICC) > 0.90, considered excellent, for both Lf and Tm. Finally, the assessment of inter-operator reliability was accounted for by repeating a scanning of the VL muscle on the same subject [24]. The following muscles were assessed, and specific evaluations were performed with the patient in a supine position and on the non-injured leg: VL, rectus femoris (RA), and tibialis anterior (TA), as well as the dominant forearm (FA) (Figure 1). Images were then exported, and “ImageJ” software (version 1.54g) was used for offline measurement of Tm (cm), Lf (cm), and θ (°), as well as area (cm^2^). These US-derived parameters were calculated as previously reported [4,16] and are summarized in Table 1. 

VL thickness and Lf were measured as previously recommended by Narici et al. [16]: The transducer was positioned at the distal third of the VL muscle, approximately 35% of its length defined by the line passing between the caudal portion of the greater trochanter and the distal border of the lateral femoral condyle. Lf was calculated between the insertions in the superior and deep aponeurosis of the fascicle, while the Tm was calculated between the orthogonal distance of the deep and superficial aponeurosis. Where the Lf extended beyond the field of view of the instrument, “ImageJ” was used to extrapolate Lf as previously recommended [16]. RF thickness was assessed at the halfway point between the epicondylus lateralis and trochanter major of the femur. The transducer was placed perpendicularly to the long axis of the thigh with adequate use of contact gel and minimal pressure to avoid excessive compression of the muscle [16]. In the same spot, by turning the transducer by 90°, we were able to measure Lf, with the use of “ImageJ” software where needed, and the θ of the muscle fibers [25]. TA scan images were taken at 30% of the distance between the head of the fibula and the tip of the lateral malleolus, measuring Tm and area [25]. The distance from the head of the fibula to the tip of the lateral malleolus was measured using a measuring tape. Forearm Tm was evaluated at the lateral forearm, 30% proximal between the styloid process and the head of the radius [26]. The USI was derived from the ultrasound images of the VL, as the ratio of fascicle length to muscle thickness (Lf/Tm) [16]. We evaluated the z-score distribution of the USI of our population (IW and WA) based on the data from a young control (YC) group as previously reported [16], i.e., 3.70 ± 0.52, using the following formula: USI Zscore = USI value − mean USI YC/SD USI YC. Sarcopenia levels based on USI z-score were defined as follows: 0.00 < z-score ≤ 1.00, non-sarcopenic subjects; 1.00 < z-score ≤ 2.00, pre-sarcopenia; 2.00 < z-score ≤ 3.00, mild sarcopenia; 3.00 < z-score ≤ 4.00, full-blown sarcopenia; z-score > 4.00, severe sarcopenia.

We finally calculated the prevalence of sarcopenia in our patients using proposed criteria based on the data collected in this study: grip strength (handgrip), with sarcopenia index for values <27 kg in males and <16 kg in females, and USI z-score, considering values >2 indicative of sarcopenia in both sexes [1]. Physical performance was not analyzed because of the presence of PFF.

### 2.3. Statistical Analysis

All statistical analyses were performed with SPSS v.22 (IBM Inc.) software. The Shapiro–Wilk test for normality of distribution was performed. In case of a non-normal distribution of the data, a log transformation was applied before further analysis. Data are reported as the means and standard deviations or counts and proportions (%) as appropriate. An independent-sample *t*-test was performed to assess differences in the reported outcomes between IW and WA, and the chi-square test was used for categorical variables such as sex. Correlation analysis with Pearson’s coefficient was performed between the anthropometric characteristics, handgrip strength, and ultrasound-derived parameters. Statistical significance was set at *p* < 0.05 for all statistical analyses, and Cohen’s *d* was reported as a measure of effect size, interpreted as small (0.2), medium (0.5), and large (0.8) [27].

## 3. Results

**Figure 2:**
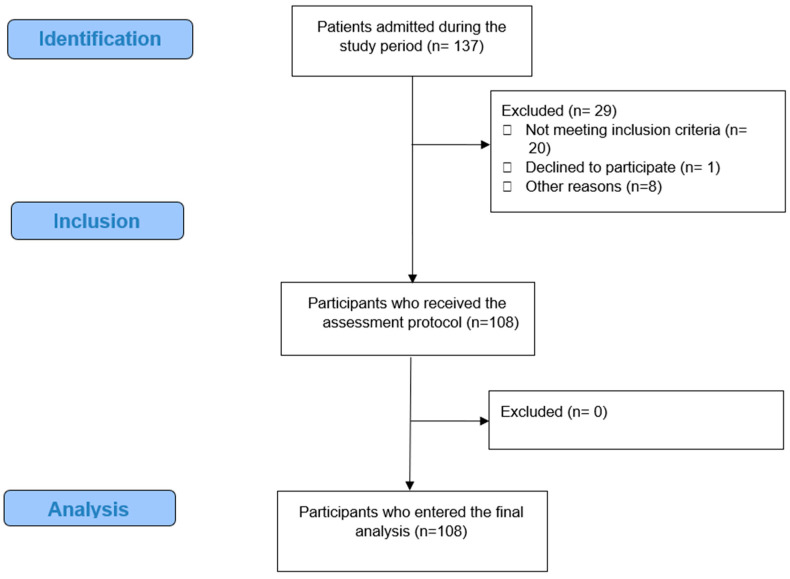
STROBE participant flow diagram.

A flowchart of study recruitment is reported in Figure 2. 

A total of 108 patients were included and without missing data. Fifty-six patients were able to walk without assistance or walking aids before trauma (IW; 83 ± 6 y; 38 females and 18 males), whereas 52 patients needed assistance or walking aids for ambulation before the trauma (WA; 87 ± 7 y; 44 females and 8 males). The WA group was characterized by older subjects (*p* = 0.002, Cohen’s *d* = 0.975) and a higher prevalence of females (*p* = 0.042). Study outcomes are reported in Table 2.

Handgrip strength was found to be 10.0 ± 3.1 kg in females and 16.2 ± 6.7 kg in males from the IW group, whereas in the WA group, we found 7.3 ± 3.7 kg in females and 9.1 ± 3.4 kg in males. Handgrip strength was found to be significantly lower by 4.5 kg (95% CI: 2.6–6.3) in the WA group compared to the IW group (*p* < 0.001, Cohen’s *d* = 0.975).

From the US evaluation, FA Tm was found to be significantly lower by 0.19 cm (95% CI: 0.04–0.33) in the WA group compared to the IW group (*p* = 0.014, Cohen’s *d* = 0.430). Although both VL Tm and Lf were significantly reduced in the WA group compared to the IW group (*p* = 0.001, Cohen’s *d* = 0.711; *p* = 0.013, Cohen’s *d* = 0.427, respectively), the USI score was higher by 1.1 (95% CI: 0.2–1.9) (*p* = 0.013, Cohen’s *d* = 0.489) in the WA group compared to the IW group. Thus, the USI z-score was higher in the WA group (3.4 ± 5.3) than in the IW group (1.4 ± 2.8) (*p* = 0.013, Cohen’s *d* = 0.471), which was suggestive of sarcopenia and pre-sarcopenia, respectively, in the two groups. The prevalence of sarcopenia was 37.3%, with values of 38.5% in males and 36.9% in females, without a significant difference for sex (*p* = 0.886).

MNA was found to be greater in the IW group (13 ± 1.5) compared to the WA group (11.4 ± 2.1) (*p* < 0.001, Cohen’s *d* = 0.876); similarly, the ADL score was found to be greater in the IW group (11.1 ± 1.8) than in the WA group (8.9 ± 2.7) (*p* < 0.001, Cohen’s *d* = 0.958). A significant correlation was found between USI and handgrip values (r = −0.207; *p* = 0.038), RF Tm (r = −0.241; *p* = 0.011) and θ (r = −0.444; *p* < 0.001), VL Tm (r = −0.766; *p* < 0.001), MNA (r = −0.267; *p* = 0.006), ADL (r = −0.222; *p* = 0.020), TA muscle area (r = −0.311; *p* = 0.001), FA Tm (r = −0.243; *p* = 0.011), forearm circumference (r = −0.193; *p* = 0.043), arm circumference (r = −0.395; *p* < 0.001), leg circumference (r = −0.394; *p* < 0.001), and thigh circumference (r = −0.397; *p* < 0.001). In addition, ADL score was found to be significantly correlated with handgrip strength (r = 0.381; *p* < 0.001), MNA (r = 0.518; *p* < 0.001), and forearm thickness (r = 0.281; *p* = 0.003).

Finally, sex differences were found when considering the whole sample, as males were characterized by higher handgrip strength (*p* < 0.001, Cohen’s *d* = 1.066); RF Tm (*p* < 0.001, Cohen’s *d* = 0.952); VL Lf (*p* < 0.001, Cohen’s *d* = 0.875) and Tm (*p* = 0.041, Cohen’s *d* = 0.492); TA area (*p* < 0.001, Cohen’s *d* = 0.771); FA Tm (*p* < 0.001, Cohen’s *d* = 1.062); and circumference of the arm (*p* = 0.008, Cohen’s *d* = 0.637), leg (*p* = 0.009, Cohen’s *d* = 0.591) and forearm (*p* < 0.001, Cohen’s *d* = 0.976). However, no significant differences were present for USI, MNA, and ADL.

## 4. Discussion

It is commonly accepted that the traditional gold standard methods for assessing muscle mass are MRI, computed tomography (CT), and DXA [1]. However, they have been reported to be often infeasible due to their limited availability and high costs, especially in orthopedic settings and in cases of prior surgery for femur fracture [28]. For these reasons, the use of ultrasound has recently been recommended for assessing muscle quantity and quality in the absence of other investigative methods, due to its good correlation with other imaging techniques and its easy applicability in the clinical setting [28]. It does not involve radiation exposure, does not require long measurement times, and can be performed at the patient’s bedside.

In particular, regarding the USI, the fundamental advantage of using a ratio (Lf/Tm), rather than absolute measures of muscle cross-sectional area or muscle mass/volume, is that it makes the measurement independent of sex, body mass, and height [16]. This new biomarker is based on a change in muscle geometric proportions due to a greater decrease in Tm than in Lf and enables us to obtain an objective diagnosis of muscle atrophy, which is essential for the classification of sarcopenia [16]. The prevalence of sarcopenia in our study (37.3%) was in line with the values found by Narici et al. (37.5%) [16] and Rustani et al. (38.7%) [8], thus confirming the similarities in our study’s population. In our study, the observed USI values were comparable to those reported in the study by Narici et al. [16], despite considering only two patient subgroups (IW and WA), as opposed to the three categories (moderately active—MAE; sedentary—SE; mobility impaired elderly—MIE) in their study. Narici et al. obtained their Z-score of the USI values by comparing the USI values of the elderly population with a young control group (30 males and 30 females all between 19 and 32 years old) [16]. We calculated the Z-score utilizing the same “control group” that Narici et al. used for their study to provide comparable findings. The WA group from this study could be considered as a combination of the subgroups labeled SE and MIE. Indeed, the WA group had a mean z-score of 3.4 ± 5.3 (compared to 2.51 of the SE group and 4.96 of the MIE group in Narici et al.’s study). In contrast, the IW group presented a z-score of 1.4 ± 2.8, which was in line with MAE. Several studies have pointed out that it is possible to assess muscle mass by performing ultrasound measurements of certain muscles, such as the RF, VL, TA, and forearm. Rustani et al.’s study proposed cutoffs for diagnosing sarcopenia based on RF Tm measured by US: 0.7 cm for females and 0.9 for males [8]. In the IW group (pre-sarcopenic according to USI), RF Tm was 0.75 ± 0.24 cm in females and 0.97 ± 0.29 cm in males, which is in line with the proposed cut-offs (>0.7 cm in females and >0.9 cm in males), whereas in the WA group (sarcopenic according to USI), RF Tm was 0.60 ± 0.21 cm in females and 0.82 ± 0.36 cm in males (<0.7 cm in females and <0.9 cm in males). 

Our results showed a significant correlation between USI and the VL thickness, but not with the fascicle length. If changes in muscle architecture were to scale harmonically with the decrease in muscle volume due to sarcopenia, one would expect the ratio of Lf to Tm to remain constant. This mirrors what was reported in previous studies [4,16]. Even though Lf decreases with age, this effect should be limited by the proximal and distal tendon insertions into bone [4]. For this reason, muscle mass reduction with aging is more related to a decrease in Tm than in Lf, which was confirmed as with an increase in the degree of sarcopenia, the decrease in Tm exceeded that in Lf [16]. Our study also revealed a statistically significant correlation between the Ultrasound Sarcopenia Index (USI) and patient autonomy before trauma, assessed both by the activity of daily living (ADL) scale and by ambulation status with (WA) or without (IW) the use of aids. In addition to this, a correlation was found with the MNA. Nutrition, in particular, is in line with studies in literature suggesting that the MNA score was significantly associated with diagnosed sarcopenia and severe sarcopenia among elderly outpatients of community hospitals [29], as well as with the study by Liguori et al. in which the MNA score was significantly lower in subjects with sarcopenia than in those without sarcopenia and the MNA score progressively decreased as muscle mass and strength reduced [30].

A noteworthy correlation was found between USI and forearm thickness and between USI and TA muscle. Regarding the TA, a significant correlation between the USI and the area of the tibialis anterior muscle was found, but a significant correlation was not found with the thickness of the tibialis anterior muscle, which is instead one of the criteria that is frequently considered in studies in the literature [24,25,28]. In addition, anthropometric measurements, in particular, calf circumference, are markers frequently used as primary screening markers for sarcopenia, in the absence of more sophisticated diagnostic methods [1]. Nevertheless, in our study, there was a significant correlation between USI and limb circumferences (forearm, arm, thigh, leg).

Furthermore, the significant correlation between USI and handgrip values reflects the already known knowledge concerning the reduction in muscle mass and strength, and consequently in physical performance, that occurs with aging and is the basis of the pathophysiological mechanism of sarcopenia [16]. This, in association with the other results in our study, makes USI potentially an excellent marker for the identification of sarcopenic patients and the optimization of their hospital management. This becomes even more important when referring to the association between sarcopenia and osteoporosis, which is an extremely frequent condition in orthopedic elderly subjects and particularly in hip fractures [3]. These reflections lead to several future perspectives as regards the use of ultrasound and USI, which can make sarcopenia conditions more easily recognized. At the same time, they can improve the impact on patients’ frailty and guide healthcare providers in choosing the most appropriate personalized treatment to optimize care, mitigate patients’ functional decline, and consequently improve overall postoperative outcomes.

According to the European Consensus on Definition and Diagnosis of Sarcopenia [1], it is necessary to assess muscle strength as well as muscle quantity and quality. In clinical practice, muscle quantity and quality are often challenging; the USI provides a “signature of sarcopenia” based on changes in muscle geometric proportions [16]. Based on the results from the present study, it might be speculated that the USI could support the stratification of elderly subjects with PFF according to the presence and severity of muscle sarcopenia. In the future, this index could be used not only as a support for the classification of sarcopenia but also as a screening tool for primary and secondary prevention. In these terms, sarcopenia might have a critical role as an important prognostic factor for frailty in this patient population [10]. Indeed, the USI could serve as a valuable tool in assessing the potential for post-surgical recovery and functional outcomes. Furthermore, the incorporation of the USI into preoperative assessments may provide clinicians with an objective and quantifiable measure for identifying individuals at higher risk of post-surgical complications and reduced functional capacity. This knowledge can aid in developing personalized rehabilitation programs and targeted interventions aimed at preserving and improving muscle strength, ultimately enhancing the overall quality of life for patients recovering from frailty proximal femur fractures.

### Limitations and Future Perspectives

It is crucial to acknowledge the limitations of this work, including the limited sample size and the heterogeneity of the included population which was representative of an ecologically valid study. Further prospective studies with larger sample sizes and rigorous methodology are warranted to validate and expand upon our findings. Additionally, the generalizability of our findings to broader populations should be considered, as our study focused specifically on patients with frailty-related fractures. Another potential limit of the study might be that some of the data was obtained after surgery, albeit within the 48 h threshold. This is due to the local guidelines for proximal femur fractures at our hospital which force us, if the patient’s health condition allows us, to perform surgery within 48 h from admission. Nevertheless, only nine participants from this study were assessed after surgery. In this study, it was not possible to collect multiple measurements at different periods from admission to monitor the progression of sarcopenia as assessed by the USI in relation to the clinical course and functional recovery. However, it was possible to show that this protocol is feasible in most clinical settings, and future longitudinal studies are encouraged. By combining the USI with clinical assessments, researchers can explore the synergistic value of ultrasound imaging in monitoring muscle mass and quality, as well as its correlation with patients’ overall clinical status. This integration could enhance the precision of prognostic predictions and guide personalized interventions to optimize recovery and mitigate the risk of frailty. Additionally, advancements in ultrasound technology, such as the development of automated algorithms for analyzing muscle characteristics and the refinement of imaging protocols, could further enhance the utility of the USI as a follow-up tool. These technological advancements, combined with comprehensive clinical assessments and the introduction of new tools such as muscular densitometry, which was not available in our study, have the potential to provide a more holistic understanding of patient recovery and guide evidence-based decision-making in post-operative management.

## 5. Conclusions

From these preliminary results, it is possible to suggest the feasibility of the USI in the clinical setting, with advantages consisting of the rapid application and low cost, providing an objective non-invasive assessment of skeletal muscle characteristics promoting a precision medicine approach. In elderly orthopedic patients, a significant correlation between the USI and other traditional markers of sarcopenia as handgrip, limb circumference, and US measurements in other muscles was found, leading to a prevalence of USI-based index of sarcopenia of 37.3%, with no significant differences between the sexes. In addition, significant differences were found according to walking independence before the fracture. In particular, the elderly requiring walking aids before hospital admission were characterized by reduced handgrip strength, forearm muscle thickness, and worse USI as measured on the vastus lateralis. Although the diagnosis of sarcopenia requires several assessments and further studies are required to better define the precision of the USI in detecting sarcopenia, the addition of ultrasound to the evaluation of older adults with fractures may help with risk stratification. In particular, it would bring significant benefits in terms of preventive medicine, identifying those subjects at a higher risk of complications and mortality according to validated markers of frailty. 

## Figures and Tables

**Table 1:** Definition of the ultrasound parameters for muscle architecture evaluation.

US Parameter	Description
Muscle thickness (Tm)	The orthogonal distance between the deep and superficial aponeuroses
Fascicle length (Lf)	The length of the fascicular path between the insertions of the fascicle into the superior and deep aponeuroses
Pennation angle (θ)	The angle of insertion of muscle fiber fascicles into the deep aponeurosis
Ultrasound Sarcopenia Index (USI)	The ratio of fascicle length to muscle thickness (Lf/Tm)

**Table 2:** Strength and ultrasound outcome assessment in the included sample. Data presented as means ± standard deviations.

	IW *n* = 56	WA *n* = 52	Significance (Cohen’s *d*)
Handgrip, kg	12.0 ± 5.4	7.6 ± 3.4	**<0.001 (0.975)**
*Circumferences, cm*			
forearm	21.4 ± 2.2	21.2 ± 2.7	0.332 (0.081)
arm	25.0 ± 2.7	24.7 ± 4.3	0.647 (0.083)
leg	30.7 ± 3.3	30.3 ± 3.5	0.586 (0.117)
thigh	41.0 ± 4.5	41.1 ± 6.6	0.869 (0.017)
*Rectus Femoris*			
thickness, cm	0.82 ± 0.27	0.64 ± 0.24	0.724 (0.704)
Lf, cm	4.79 ± 0.82	4.52 ± 0.84	0.114 (0.325)
pennation angle, °	9.9 ± 2.6	8.8 ± 3.5	0.054 (0.356)
*Vastus Lateralis*			
thickness, cm	1.27 ± 0.32	1.02 ± 0.38	**0.001 (0.711)**
Lf, cm	5.26 ± 0.87	4.89 ± 0.86	**0.013 (0.427)**
*Tibialis Anterior*			
thickness, cm	1.78 ± 0.29	1.60 ± 0.40	0.313 (0.515)
area, cm^2^	5.3 ± 1.4	4.8 ± 1.7	0.114 (0.321)
*Forearm*			
thickness, cm	1.37 ± 0.40	1.20 ± 0.39	**0.014 (0.430)**
*USI*			
Score	4.4 ± 1.5	5.5 ± 2.8	**0.013 (0.489)**
z-score	1.4 ± 2.8	3.4 ± 5.3	**0.013 (0.471)**

Notes: IW: independent walking; WA: walking aid; USI: Ultrasound Sarcopenia Index; Lf: fascicle length. Independent-sample *t*-test, bold values for *p* < 0.05 (effect size, Cohen’s *d*).

## Data Availability

The data presented in this study are available on request from the corresponding author. The data are not publicly available due to data protection regulation from this institution.
